# Severe axial vertebral rotation treated with a modified Boston brace: a case report

**DOI:** 10.1186/1748-7161-5-5

**Published:** 2010-03-24

**Authors:** Eustathios I Kenanidis, Michael E Potoupnis, Kyriakos A Papavasiliou, Fares E Sayegh, George A Kapetanos

**Affiliations:** 1 3rd Orthopaedic Department, Aristotle University of Thessaloniki-Medical School, "Papageorgiou" General Hospital of Thessaloniki, Greece

## Abstract

We report the case of a 13-year-old Caucasian girl suffering from severe axial rotation of the T5 to L4 vertebrae. The patient (initially examined during a school screening study) was at first considered to be suspicious of suffering from scoliosis due to a highly positive Adam's forward bending test. However, her radiographic evaluation revealed the existence of axial rotation in 12 of her vertebrae, without inclination in the sagittal and coronal planes. After an observation period of 12 months and due to the fact that both her physical appearance and the measured vertebral rotation deteriorated, the patient was given a modified thoracolumbar Boston brace that had an immediate positive derotational effect on all but two vertebrae. Twenty four months later, the progress of the vertebral rotation(s) seems to have been halted and most affected vertebrae appear to be stabilized in their new, 'post-brace', reduced position, with better results shown when the Boston brace is worn. The patient remains under constant medical observation. The application of a modified Boston brace seems to have served well (so far) a useful purpose for reducing and stabilizing this case of severe axial vertebral rotation, providing less deformity and (possibly) offering a better final cosmetic result.

## Background

Adolescent Idiopathic Scoliosis (AIS) is a complex three-dimensional deformity of the spine [1]. The normal, non-scoliotic spine on the other hand demonstrates a pre-existent pattern of minimal axial vertebral rotation that somewhat corresponds to what is seen in the most prevalent types of thoracic idiopathic scoliosis [2]. It is rather uncommon [3] for a patient to be diagnosed with a condition (such as vertebral rotation in the horizontal plane without inclination in the sagittal and coronal planes) that seems to lie somewhere 'in the middle' between a normal and a scoliotic spine. This clinical entity is usually accompanied by minimal clinical deformity and (although rarely) it can interfere with the results of scoliosis screening studies, often leading to a false diagnosis of scoliosis and unnecessary radiographic examination [3]. Whether this deformity is an independent clinical entity or a precursor to structural scoliosis is currently not well understood [4].

Boston brace has been widely used for the treatment of children suffering from AIS [5-7]. This brace seems to beneficially affect the natural history of scoliotic children [5] with moderate influence on their quality of life [6,7]. However, no such treatment has ever been reported for patients suffering from axial vertebral rotation in the horizontal plane.

According to the best of our knowledge, this is the first report of a patient suffering from axial vertebral rotation in the horizontal plane, who was treated with the application of a modified Boston brace. Aim of this study is to remind the reader that, although it is not very frequent, axial vertebral rotation does occur, it is often misdiagnosed and/or may lead to a false diagnosis of scoliosis. Furthermore, it is not uncommon for this clinical entity to be accompanied by substantial physical deformity; hence a careful evaluation of all patients suffering from this deformity must always be performed.

## Case presentation

We report the case of a 13-year-old Caucasian girl suffering from axial rotation of the T5 to L4 vertebrae in the horizontal plane. The patient was otherwise fit and well, her reported medical history was free and both she and her parents were not familiar with any cases of scoliosis among members of her family. The patient (initially examined during a school screening study-Visit 1) was considered at first to be suspicious of suffering from scoliosis, due to a highly positive Adam's forward bending test. However, radiographic evaluation revealed the existence of axial vertebral rotation (Grades 1-2 according to the Nash and Moe method of radiographic assessment) [8] without any deformity whatsoever in the sagittal and coronal planes (Table 1). Her physical (and radiographic) examination failed to reveal the existence of any discrepancy in her limbs' length or any other skeletal abnormality. Due to the unfortunate fact that the patient shortly after her initial examination lost all her radiographs and in order to better evaluate both the structural deformity underlying the vertebral rotation and the possible co-existence of a thoracic cage deformity which could interfere with the interpretation of the Adam's forward bending test, a Computed Tomography (CT)-scan was suggested. Unfortunately, both the patient and her parents refused to consent to it.

**Table 1:** The rotational radiographic direction and deformity of each affected vertebra.

		**Vertebral Rotation as measured by the Nash and Moe method **[8]				
		
Vertebra	Rotational Direction	Visit 1 Initial Examination	Visit 2 12 months after initial examination		Visit 3 36 months after initial examination	
		
			No Brace	With Brace	No Brace	With Brace
**T5**	Right	1	1-2	0	1	0

**T6**	Right	1	1-2	0	1	0

**T7**	Right	1	2	1	1	1

**T8**	Right	1	2	1	2	1

**T9**	Right	2	2-3	1	2	1

**T10**	Right	2	2-3	1	2	1

**T11**	Right	1	2	1	2	1

**T12**	Right	1	2	1	2	1

**L1**	Right	1	2	1	2	1

**L2**	Right	1	2	1	2	1

**L3**	Right	1	2	1	2	1

**L4**	Right	1	1	1	1	1

**Figure 1:**
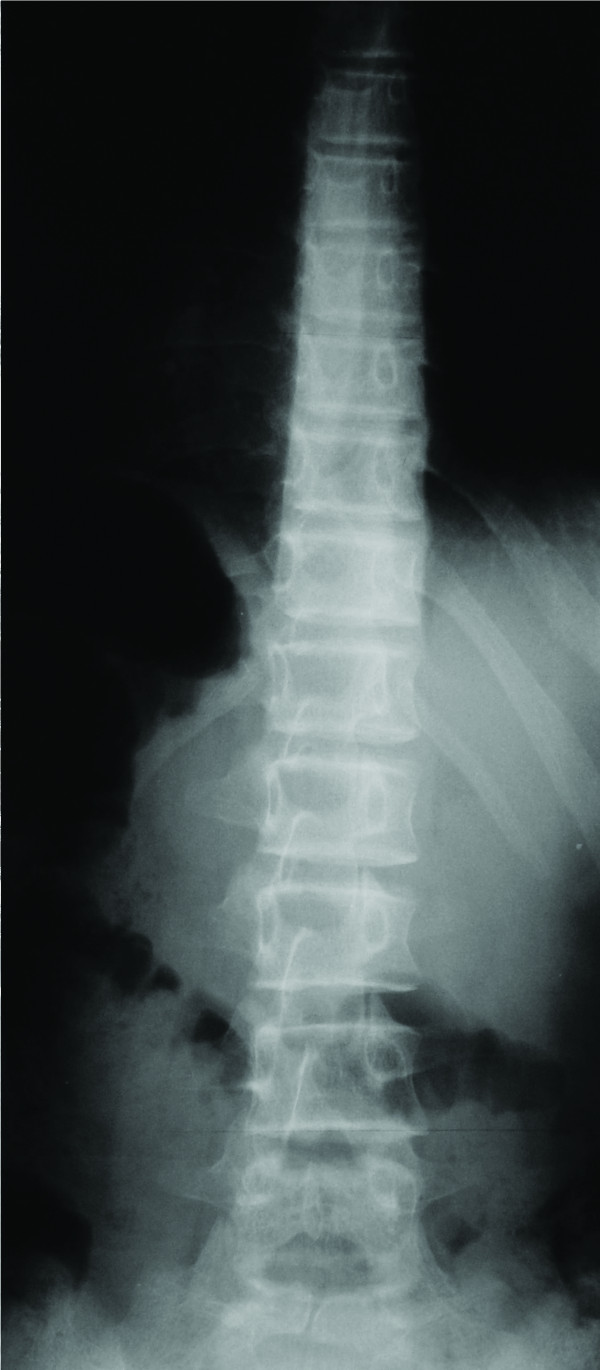
**Standing posterior-anterior radiograph of the patient's spine 12 months after her initial examination (Visit 2)**. The vertebral rotation has significantly deteriorated leading to the decision to fit the patient with a modified thoracolumbar Boston Brace.

**Figure 2:**
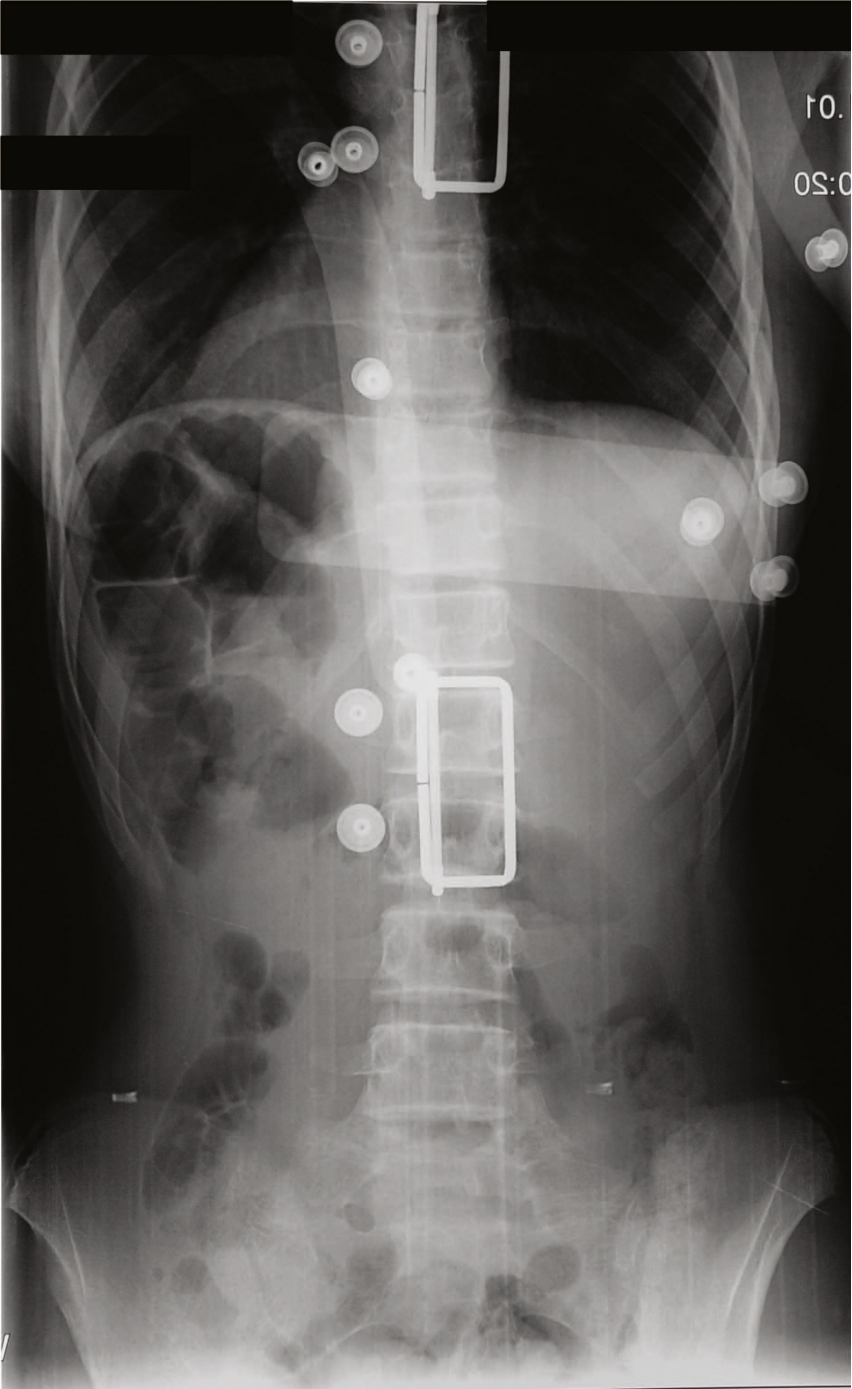
**Standing posterior-anterior radiograph of the patient's spine following the application of a modified Boston brace (Visit 2)**. Notice the immediate derotational effect of the brace.

Following an observation period of 12 months the patient was re-evaluated (Visit 2). Due to her young age, the recent reported menarche at the age of eleven (between Visits 1 & 2), her skeletal immaturity (Risser stage I-II), the fact that both the patient and her parents reported that the appearance of the patient's trunk worsened and the fact that the measured vertebral rotation deteriorated (Table 1) (Figure 1), the patient was given a modified Boston brace (fitted with derotational blades) which appeared to have had an immediate positive derotational effect on all but two vertebrae (Figure 2). The patient was instructed to wear the brace for 16 hours daily; however the mean time of brace application was probably about fourteen hours daily.

**Figure 3:**
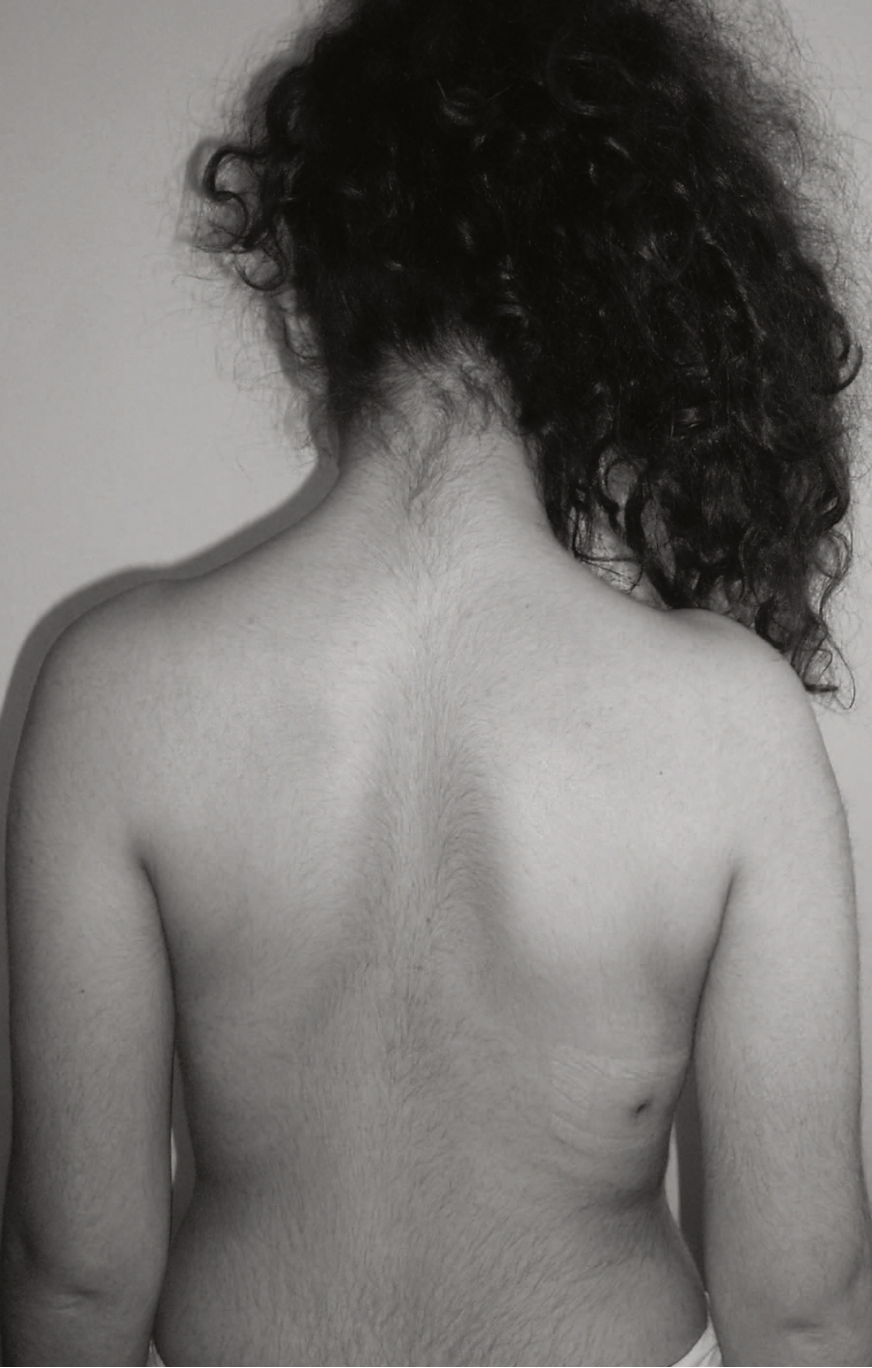
**The patient upon her latest follow-up visit (Visit 3), observed in the standing erect position-dorsal view**. Notice the skin irritation that developed due to the brace application which is being treated conservatively.

**Figure 4:**
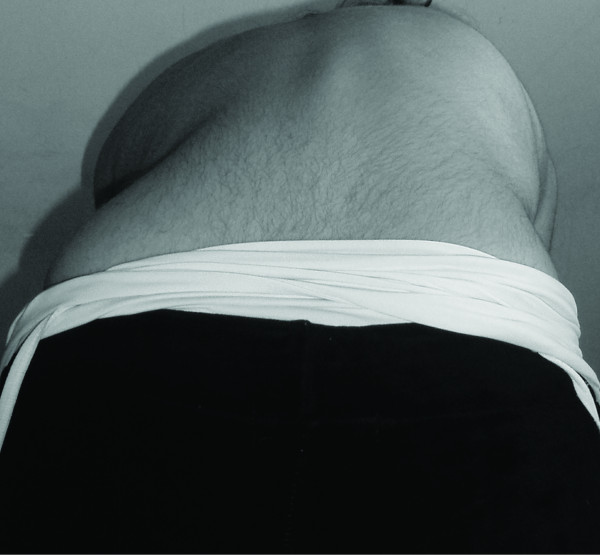
**The patient upon her latest follow-up visit (Visit 3), performing the 'Adam's bending test**. Notice the still clinically apparent (despite the significant de-rotational effect of the brace) hump at her right side due to the severely horizontally rotated T5-L4 vertebrae.

**Figure 5:**
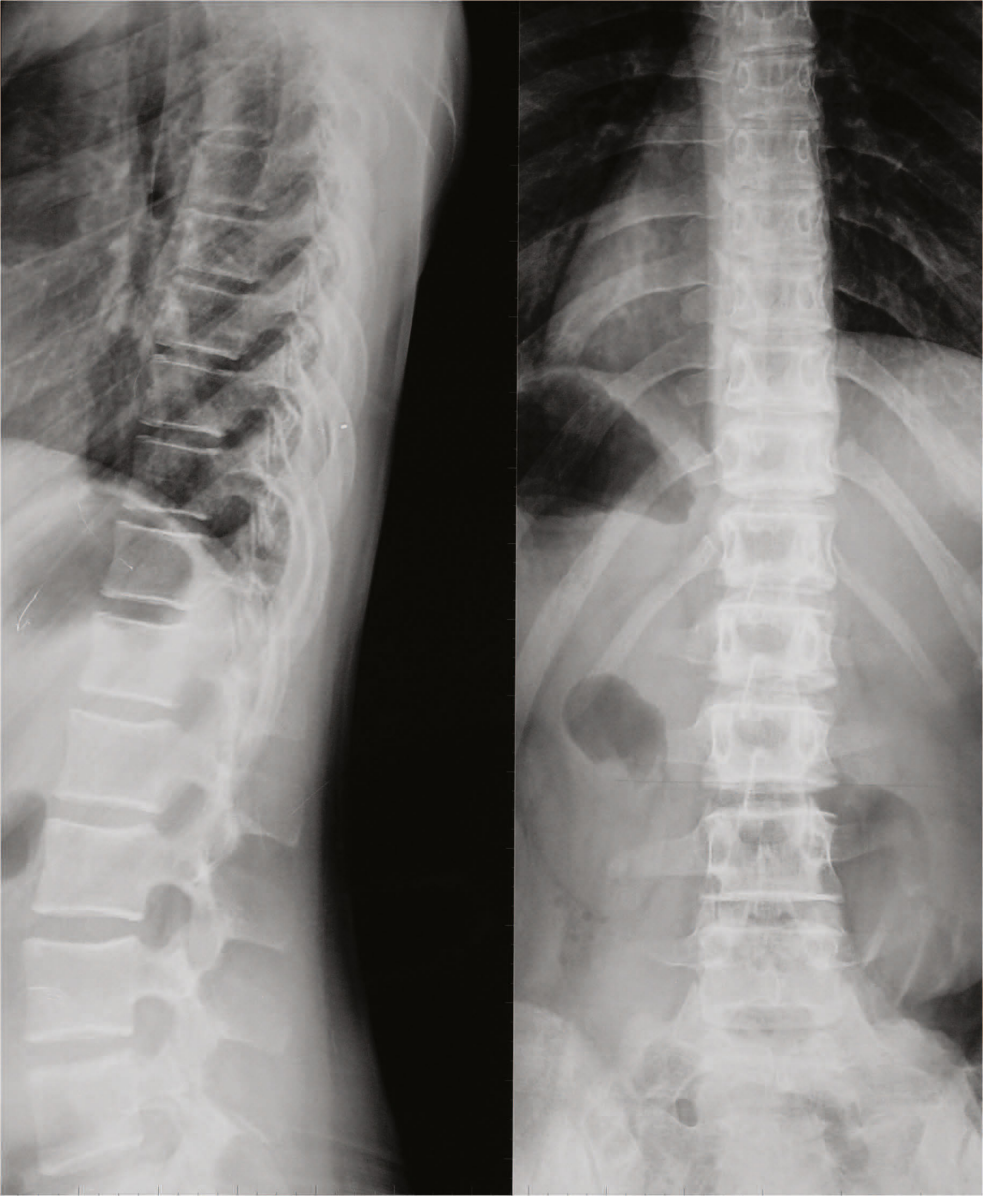
**Standing (brace not worn) posterior-anterior and lateral radiographs 24 months after the initial application of the brace (Visit 3)**. The vertebral rotation has significantly improved in most of the affected vertebrae.

**Figure 6:**
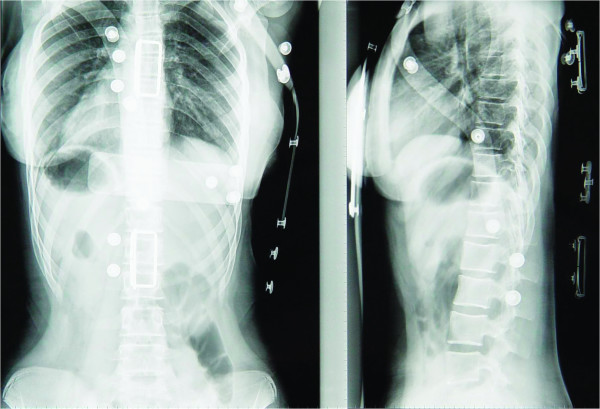
**Standing (brace worn) posterior-anterior and lateral radiographs 24 months after the initial application of the brace (Visit 3)**.

Twenty four months later at Visit 3 (Figures 3, 4), the progress of the vertebral rotation(s) seems to have been halted and most affected vertebrae appear to have been stabilized in their new, 'post-brace', reduced position (Figure 5). As expected, better results are radiographically apparent when the Boston brace is worn (Table 1) (Figure 6). The patient is under constant medical observation (Risser stage IV upon her latest follow-up Visit 3); she is quite happy with the result of her therapy (as she reports that the rib-hump has -to some extent- been reduced in size) and remains symptoms-free, apart from a skin irritation caused by the brace which is being treated conservatively (Figure 3).

## Discussion

Adolescent idiopathic scoliosis is a complex three-dimensional deformity of the spine characterized by thoracic lordosis in the sagittal, lateral curve(s) in the coronal and vertebral rotation in the transverse plane [1]. The horizontal rotation of the vertebral bodies is a well-recognised, essential and typical component of AIS [2,4], even though both its exact pattern [3] and its impact on the development of a clinically apparent hump [9], are sometimes the subject of controversy. On the other hand, axial vertebral rotation in the horizontal plane is a significantly less common albeit well acknowledged clinical entity [3].

Armstrong et al. [3] who initially described this deformity, reported 4% of patients with an asymmetric Moiré topography being diagnosed with axial vertebral rotation in the horizontal plane. It appears that either lateral deviation of the spine, axial vertebral rotation or thoracic cage deformities [10], can equally lead to the development of a clinically noticeable hump, although there seems to be a higher correlation between vertebral rotation (when compared with lateral curvature) and Moiré hump detection [3]. Apparently, in patients that do have a hump but clinically their spine do not deviate from the midline, axial vertebral rotation and not the scoliotic curve is the cause of their deformity [3]. Axial vertebral rotation is often associated with minimal clinical deformity, which nonetheless may lead to a false diagnosis of scoliosis [3]. More severe types of this deformity however do exist and are certainly bound to 'produce' a clinically more apparent rib-hump and interfere with the interpretation of the Adam's forward bending test and the subsequent diagnosis of scoliosis. A positive Adam's test may be apparent in patients with thoracic cage deformity alone. In this case the patient's "rib-index' is >1 [10,11]. Even though our patient's 'rib-index' was >1 in all affected levels/vertebrae, we believe that this must be attributed to the vertebral rotation and not to the existence of a thoracic cage deformity, which was neither clinically, nor radiographically apparent. However, it is true that a CT-scan would have identified beyond any doubt any co-existing rib-cage deformity. Unfortunately the patient did not consent to it.

The typical curves in the sagittal and coronal planes together with the coexisting axial rotation in the horizontal plane pattern have been well described in patients suffering from AIS [2]. However, the possible patterns of rotation in the normal, non-scoliotic spine have been addressed in very few studies so far [2,4,12]. Kouwenhoven et al. [2] showed that axial rotation in the normal, non-scoliotic spine was not neutral or randomly distributed and was characterised by a predominant rotation to the left of the high thoracic vertebrae (T1-T4), and to the right of the mid and lower thoracic (T5-T12) and lumbar vertebrae. This rotation pattern differed significantly from an equal right-left distribution. When males and females were analyzed separately, rotation remained significant to the right in the mid and lower thoracic region; in the high thoracic region however, rotation to the left was significant only in female patients. Our patient's vertebral rotational pattern seems to be in agreement with these findings, as all involved vertebrae (T5-T12 and L1-L4) are rotated towards the right side (Table 1).

The natural history and course of this deformity remains more or less unknown [3]. Whether it is an independent clinical entity or a precursor to structural scoliosis (in its more severe forms?) is currently not well understood [4]. However, the relatively high incidence of minimally 'deformed' axially rotated vertebrae found in the spine of practically all normal adults [2], may potentially suggest the existence of a 'self-limited' or even 'self-remitting' pattern, especially in patients with minor deformities.

Whether axial vertebral rotation is amenable to treatment or not is another interesting question. Grivas et al. [5] supported that the modified Boston brace treatment alters the natural history of the Adolescent Idiopathic Scoliosis. The authors also proposed some aetiological implications on the deforming forces and the likelihood of neuromuscular factors involved in its aetiology. Van Rhijn et al. [13] showed that AIS is a dynamic process, hence the type of curve in patients suffering from AIS can change due to external influences such as brace treatment. Furthermore there is evidence that the application of a brace does not stop progression of the lumbar curve as well as it stops progression of the thoracic curve [13,14]. The latter may be the end-result of the better transmitted (through the rib-cage), externally applied corrective forces of the brace to the thoracic vertebrae. Our patient's result seems to support this theory, as the application of a modified Boston brace for a period of two years seemed to have had a better (radiographically assessed) derotational effect on the thoracic vertebrae (either with or without the brace) when compared with the achieved derotation of the lumbar vertebrae (especially without the brace) (Table 1). The truth is that the influence of brace treatment on both the vertebral rotation [15,16] and the clinical appearance of the rib-hump deformity [17] of patients suffering from AIS, is still a rather controversial issue, even though increased rotation seems to be an independent negative prognostic factor [18].

Although Boston brace has been widely used for the treatment of children suffering from Adolescent Idiopathic Scoliosis [5], with moderate influence on their quality of life [6,7], the use of such a treatment has never been proposed for patients suffering from axial vertebral rotation in the horizontal plane and is certainly not currently recommended by the SRS. The decision to start treating our patient was not easy. Following a period of observation of twelve months, which apparently leaded to significant clinical (as self-assessed by the patient and her parents) and radiographic deterioration, we decided to implement a more aggressive approach; hence we proposed the application of a modified Boston Brace, regardless of the fact that no spinal deformity was identified in the sagittal and coronal planes. This decision was mainly based on the hypothesis that by wearing it until skeletal maturity was reached, a derotational [19] and subsequently a (possibly) better cosmetic effect could be expected, even though the efficacy of Boston Brace in this field is another controversial issue [15,16]. Although the exact mechanism by which derotation is achieved is not yet fully clarified, it is postulated that the exerted by the brace continuous pressure on the sagittal plane which is further enhanced by the respiratory movements of the thoracic cage and the soft-tissue mediated forces, contribute to the derotational effect of the brace [20,21]. The result (so far) is promising, since Boston brace seems to have managed to halt (and in certain vertebrae even reverse) the progression of this deformity (Table 1). The short period of follow-up is certainly a limitation of our paper and we are well aware of the fact that a definite conclusion regarding the successful or not application of the Boston brace in this case will be drawn after the completion of the treatment period and the removal of the brace. A period of at least one year following the removal of the brace will allow us to judge the efficacy of the treatment.

## Conclusions

It is true that we cannot jump to final conclusions based only on the result of one patient's case, even though this seems to be extremely promising. Given however the scarcity of this deformity (at least in its severe form), it is doubtful whether large series studies that would evaluate the efficacy of the application of a Boston brace in the treatment of this deformity are feasible. This case study does not support the use of a Boston brace in order to treat every single patient suffering from axial vertebral rotation, as this is totally unnecessary in the majority of the cases, and furthermore its application is not supported by the literature. In patients however with severe deformity (that is usually accompanied by a clinically apparent and psychologically challenging hump) it might be worth trying it. Being significantly less morbid and more cost-efficient than thoracoplasty (which is certainly a 'final treatment option' for both the surgeon and the patient), the application of a modified Boston brace in the treatment of patients suffering from severe axial vertebral rotation in the horizontal plane seems to be a safe, simple, efficient and (possibly) successful, alternative to surgery treatment.

## Consent

Written informed consent was obtained from the parents of the patient for publication of this case report and any accompanying images. A copy of the written consent is available for review by the Editor-in-Chief of this journal

## Competing interests

The authors declare that they have no competing interests.

## Authors' contributions

EIK have made substantial contributions to conception and design, acquisition of data, analysis and interpretation of data. He has been also involved in drafting and revising the manuscript for important intellectual content.

MEP contributed to the conception, acquisition of data and interpretation of data. He revised it critically for important intellectual content.

KAP has made substantial contributions to the analysis and interpretation of data. He has been involved in drafting the manuscript and revising it critically for important intellectual content.

FES revised the manuscript critically for important intellectual content and gave final approval of the version to be published

GAK revised the manuscript critically for important intellectual content and gave final approval of the version to be published

## References

[B1] Deacon P Flood BM Dickson RA Idiopathic scoliosis in three dimensions. A radiographic and morphometric analysis J Bone Joint Surg Br 1984 66 509 512 6746683 10.1302/0301-620X.66B4.6746683

[B2] Kouwenhoven JW Vincken KL Bartels LW Castelein RM Analysis of preexistent vertebral rotation in the normal spine Spine (Phila Pa 1976) 2006 31 1467 1472 16741456 10.1097/01.brs.0000219938.14686.b3

[B3] Armstrong GW Livermore NB Suzuki N Armstrong JG Nonstandard vertebral rotation in scoliosis screening patients. Its prevalence and relation to the clinical deformity Spine (Phila Pa 1976) 1982 7 50 54 7071661 10.1097/00007632-198200710-00006

[B4] Sevastik B Xiong B Sevastik J Hedlund R Suliman I Vertebral rotation and pedicle length asymmetry in the normal adult spine Eur Spine J 1995 4 95 97 10.1007/BF00278919 7600157

[B5] Grivas TB Vasiliadis E Chatziargiropoulos T Polyzois VD Gatos K The effect of a modified Boston brace with anti-rotatory blades on the progression of curves in idiopathic scoliosis: aetiologic implications Pediatr Rehabil 2003 6 237 242 14713591 10.1080/13638490310001636808

[B6] Vasiliadis E Grivas TB Savvidou O Triantafyllopoulos G The influence of brace on quality of life of adolescents with idiopathic scoliosis Stud Health Technol Inform 2006 123 352 356 17108451

[B7] Vasiliadis E Grivas TB Quality of life after conservative treatment of adolescent idiopathic scoliosis Stud Health Technol Inform 2008 135 409 413 18401108

[B8] Nash CL Jr Moe JH A study of vertebral rotation J Bone Joint Surg Am 1969 51 223 229 5767315

[B9] Thulbourne T Gillespie R The rib hump in idiopathic scoliosis. Measurement, analysis and response to treatment J Bone Joint Surg Br 1976 58 64 71 1270497 10.1302/0301-620X.58B1.1270497

[B10] Grivas TB Dangas S Polyzois BD Samelis P The Double Rib Contour Sign (DRCS) in lateral spinal radiographs: aetiologic implications for Scoliosis Stud Health Technol Inform 2002 88 38 43 15456003

[B11] Grivas TB Vasiliadis ES Mihas C Savvidou OD The effect of growth on the correlation between the spinal and rib cage deformity. Implications on idiopathic scoliosis pathogenesis Scoliosis 2007 2 11 10.1186/1748-7161-2-11 17868459 PMC2040132

[B12] Xiong B Sevastik B Sevastik J Hedlund R Suliman I Kristjansson S Horizontal plane morphometry of normal and scoliotic vertebrae: a methodological study Eur Spine J 1995 4 6 10 10.1007/BF00298410 7749910

[B13] Van Rhijn LW Plasmans CM Veraart BE Changes in curve pattern after brace treatment for idiopathic scoliosis Acta Orthop Scand 2002 73 277 281 12143972 10.1080/000164702320155248

[B14] Andriacchi TP Schultz AB Belytschko TB Dewald R Milwaukee brace correction of idiopathic scoliosis. A biomechanical analysis and a retrospective study J Bone Joint Surg Am 1976 58 806 815 956227

[B15] Aaro S Burston R Dahlborn M The derotating effect of the Boston brace Spine (Phila Pa 1976) 1981 6 477 482 7302681 10.1097/00007632-198109000-00009

[B16] Labelle H Dansereau J Bellefleur C Poitras B Spine (Phila Pa 1976) 1996 21 59 64 9122764 10.1097/00007632-199601010-00013

[B17] Aubin CE Dansereau J de Guise JA Labelle H Rib cage-spine coupling patterns involved in brace treatment of adolescent idiopathic scoliosis Spine (Phila Pa 1976) 1997 22 629 635 9089935 10.1097/00007632-199703150-00010

[B18] Vijvermans V Fabry G Nijs J Factors determining the final outcome of treatment of idiopathic scoliosis with the Boston brace: a longitudinal study J Pediatr Orthop B 2004 13 143 14 10.1097/00009957-200405000-00001 15083112

[B19] Korovessis P Kyrkos C Piperos G Soucacos PN Effects of thoracolumbosacral orthosis on spinal deformities, trunk asymmetry, and frontal lower rib cage in adolescent idiopathic scoliosis Spine (Phila Pa 1976) 2000 25 2064 71 10954637 10.1097/00007632-200008150-00010

[B20] Grivas TB Vasiliadis ES Cosmetic outcome after conservative treatment of idiopathic scoliosis with a dynamic derotation brace Stud Health Technol Inform 2008 135 387 92 18401106

[B21] Castro FP Jr Adolescent idiopathic scoliosis, bracing, and the Hueter-Volkmann principle Spine J 2003 3 180 185 10.1016/S1529-9430(02)00557-0 14589197

